# Irritable mood and the Diagnostic and Statistical Manual of Mental Disorders

**DOI:** 10.1186/1753-2000-3-35

**Published:** 2009-10-24

**Authors:** Daniel J Safer

**Affiliations:** 1 Departments of Psychiatry and Pediatrics, Johns Hopkins University School of Medicine, Baltimore, Maryland USA

## Abstract

**Background:**

The terms 'irritable mood' and 'irritability' have been applied to describe and define a variety of different categories in the Diagnostic and Statistical Manual of Mental Disorders (DSM). More precise diagnostic terms and concepts are needed.

**Methods:**

A concise critical historical review of DSM categories characterized by irritability, anger, and aggression is presented followed by recommendations.

**Results:**

This analysis describes the broad ranging and imprecise use of the term irritability since the first DSM in 1952. A more age-appropriate and functional realignment of psychiatric categories linked to dysfunctional anger is suggested. Among other recommendations, this realignment would remove irritability as a problematic definer in the present DSM mood categories: expand oppositional defiant disorder to include adults; link the callous unemotional subtype of conduct disorder in adolescents to antisocial personality disorder; move intermittent explosive disorder to an appropriate category: and expand the term 'mood' to apply also to dysfunctional anger and anxiety.

**Conclusion:**

The non-specific term 'irritability' commonly used in the DSM has had an adverse effect on diagnostic specificity and thereby on treatment. Dysfunctional anger is a major mood disorder which merits a more prominent and better defined representation in psychiatric nomenclature.

## Introduction

Irritable mood, defined in the Diagnostic and Statistical Manual of Mental Disorders (DSM) [[1]^p.825^] as "easily annoyed and provoked to anger," and irritability have been part of numerous DSM diagnoses since 1952. After a presentation of diagnostic background material and relevant diagnostic terms, recommendations will be made to more precisely categorize disorders related to dysfunctional anger.

## Background on Irritable Mood in the DSM

### 1) Irritable mood has been a defining DSM characteristic of manic episodes since 1952

Irritable mood became a major *defining *characteristic of manic episodes beginning in 1952 with the first DSM [2]^p.25] ^as evidenced by the following sentence describing a manic-depressive reaction, manic type: "This group is characterized by elation or irritability and over- talkativeness, flight of ideas and increased motor activity". In DSM-III [[3]^p.208^], a manic episode required "one or more distinct periods with a predominantly elevated, expansive or irritable mood," a definition which is nearly identical in subsequent DSM revisions [[4]^p.217^, 5 ^p.328^]. Thus, irritable mood by itself can substitute for elated or expansive mood as the diagnostic *basis *for a manic or hypomanic episode.

### 2) Irritable mood is restricted to the Mood Disorders category of the DSM

Mood disorders in the DSM-IV include *only *depressive and bipolar disorders. Three different moods define these diagnoses. They are depressive, expansive/elevated, and irritable moods. Elevated/expansive mood defines manic episodes, as can irritable mood. Depressed mood exclusively defines depressive disorders, except that *in youth *an irritable mood can substitute for a depressed mood to define major depressive episodes and dysthymic disorders [1]. In the DSM--of course, to establish a diagnosis of a mood disorder beyond the presence of a defined mood, a duration criterion must be reached and a specific number of symptom descriptors are required. Pathological anxiety and anger are not considered to be mood disorders in the DSM, although they are listed as abnormal moods in a majority of psychiatric texts and dictionaries [6-8]

### 3) Irritability is a common symptom descriptor in many DSM categories

Irritability is listed as a descriptor of numerous and varied disorders in the DSM-IV. These include Generalized Anxiety Disorder, Post Traumatic Stress Disorder, Borderline Personality Disorder, Antisocial Personality Disorder, Nicotine Withdrawal, Pathological Gambling, and Schizoaffective Disorder [1,9,10]. Although irritability is not a specific descriptor in Oppositional Defiant Disorder, its importance in that disorder can be inferred from descriptors such as "often loses temper" and "easily annoyed by others". Irritability is also not included as a DSM descriptor for depressive disorders, but in reports of presenting symptoms by depressed adults, it is recorded in the majority of cases [11-13]. Similar rates of irritability (73%) were reported by adults with bipolar disorder when entering the large STEP-BD trial [14].

### 4) Aggressive behavior defines numerous personality and behavior disorder diagnoses in the DSM

In the DSM, frequent episodes of rage and aggression have defined a number of personality and problematic behavior disorders. In the first DSM [[2]^p.37^], aggression was identified as passive-aggressive personality, aggressive type. In DSM II [[15]^p.42^] explosive personality disorder was characterized by "gross outbursts of rage or verbal or physical aggressions". In DSM III, IIIR and IV, intermittent explosive disorder was characterized as aggression "...grossly out of proportion to any... precipitating psychosocial stressors..." that result in serious assaultive acts or destruction of property [[5]^p.609^]. Antisocial personality disorder diagnostic descriptors in DSM IV [[5]^p.650^] included: "...aggressiveness, as indicated by repeated physical fights or assaults" and "reckless disregard for the safety of self or others". For youth, conduct disorder was characterized by destruction of property, aggression to people and animals, and theft [[5]^p.90^]. As indicated, aggressive behavior--not irritability--defines these DSM disorders.

### 5). Terms like emotion, mood and trait are seldom clearly applied in psychiatric practice

An emotion is an aroused mental state accompanied by an autonomic manifestation that usually lasts minutes or hours and is triggered by events. A mood is a pervasive emotion lasting days and occasionally weeks and may occur without an obvious trigger. A trait is present for months or years and represents a discrete personality pattern [16,17]. Irritability is a physiological emotional response to a provoking stimulus; it may be recurrent but it is usually short lived. Anger is a cognitive reaction to an aversive circumstance, ranging from displeasure to rage; it may or may not be dysfunctional. Aggression is a behavioral or motoric response associated in psychiatry with an intent to do harm; it may be self-directed. Hostility is a directed, maintained attitude of ill-will [18-20].

## Problems with Irritability in the DSM

### A. Depression

**1) **Irritable mood was ill-conceived as a DSM diagnostic *definer *of dysthymia and major depressive disorder (MDD) in youth. Irritability is a fairly common feature in youth diagnosed with MDD, 38%--55% [21,22], and outcome studies bear out a strong relationship between irritability in youth and depression in young adulthood [23,24]. But unlike sadness and anhedonia, irritability is not a core feature of depressed mood [19,25,26].

**2) **Irritability is at least as common in depressed adults [11-13] as in depressed youth. In the large STAR*D naturalistic clinical trial, 81% of the adults with MDD entering the trial reported irritability, and half of this group reported that this symptom occurred more than half of their waking hours [13]. Nonetheless, even though irritability is very frequently reported by adults with depression, it is not listed in the DSM as a definer or a descriptor for MDD in *adulthood*. This indicates a perplexing age-group inconsistency in the DSM for the inclusion of irritability to characterize depression.

**3) **Irritability has been frequently used as a primary diagnostic feature to separate bipolar from unipolar depression [27,28]. It is indeed the case that those experiencing both MDD and irritability--in group data--have more impairment, a somewhat different family history and an increased vulnerability to stress [13]. But one needs to consider that irritability is often an associated feature of numerous other chronic psychiatric conditions--(e.g, ADHD, mental retardation, Alzheimer's disease) -and it usually adds to a patient's risk of untoward consequences. Thus, it is unclear at present if a depression with irritable mood -by itself--meaningfully constitutes a distinct diagnostic entity.

### B. Anger

**1) **Irritability, anger, defiance and temper are specific descriptors of oppositional defiant disorder (ODD) in youth. ODD is operationally distinct from conduct disorder which is characterized by behaviorally aggressive acts. In adults, diagnoses of dysfunctional anger are not as clearly delineated. Antisocial behavior disorder in the DSM is included as a personality disorder and its diagnosis focuses on violations of the rights of others [1]; it closely matches an increasingly researched subgroup of conduct disordered adolescents identified as callous--unemotional [29]. Intermittent explosive disorder (IED) is characterized by impulsive aggressive and assaultive behaviors out of proportion to stressors. IED is grouped in the DSM under Disorders of Impulse Control, Not Elsewhere Classified and combined in that category with kleptomania, trichotillomania (beginning in 1987), pathological gambling and pyromania, disorders that are more compulsive than impulsive [30]. In adults there is no category akin to ODD in youth; essentially, there is no category for adults who are frequently dysfunctionally angry but not physically assaultive or repeatedly destructive.

**2) **Dysfunctional anger is one of the three most frequently cited pathological emotions, the others being profound sadness and anxiety/fear [31]. Anger, however, is not listed in the index of the DSM IV-TR [1]. Furthermore, anger, irritability, rage and irritable mood were not defined by the American Psychiatric Glossary in 1980, 1988, 1994 and 2003 [32], and dysfunctional anger is not listed in the DSM as a possible consequence of alcohol intoxication [1,20]--a not uncommon development [33].

**3) **Fortunately, psychiatric diagnoses in the National Comorbidity Survey--Replication (NCS-R) based on *community interview data *were newly grouped into 4 major categories; anxiety, mood, impulse control, and substance abuse disorders (34). Included in the impulse control disorder group were: attention deficit hyperactivity disorder, opposition defiant disorder, conduct disorder, and intermittent explosive disorder. In the NCS-R analyses, impulse control disorders had a 24.8% lifetime prevalence in adults and a 20% twelve-month prevalence in adolescents--both second only to anxiety disorders [34,35]. Thus, disorders largely associated with dysfunctional anger are not at all uncommon in the population--even though they don't merit a high profile in the DSM.

### C. Mania

**1) **Although irritable mood in the DSM is one of the defining features of manic episodes, it is not specific for mania. Manic mood at its extreme is commonly characterized by frenzied, ungovernable exuberance--essentially elation not grounded in reality [8,36]. Acute manic episodes generally include multiple symptom dimensions characterized by accelerated speech shifting in context, increased motor activity, an expansive/elated commonly delusional outlook, decreased sleep, and often also by grandiose ideation, paranoia, dysphoria, distractibility, and irritability/anger [8]. In factor analyses, anger/aggressiveness during a manic episode has been found to be a separate dimension from elation; it is most closely linked to paranoia [37,38]. Irritability and anger are seldom initial symptoms of this disorder; they generally develop later in course of a manic episode [36,39,40].

**2**) It is quite possible to meet the DSM criteria for a manic or hypomanic episode by having a recurrent pattern of irritable mood with 4 of the 7 symptom descriptors of the disorder. These descriptors could be: more talkative than usual, distractible, decreased need for sleep, subjective racing thoughts, and psychomotor agitation [1]. If four such symptom criteria along with recurrent irritability are identified during a psychiatric interview, this could partially explain how 27%-34% of U.S. psychiatric inpatient youth received a primary discharge diagnosis of bipolar disorder in 2004 [41].

## Concluding Comments and Suggestions

Biological correlates of dysfunctional anger disorders, such as a higher androgen level, a low heart rate, and a low level of the primary CSF serotonin metabolite [42], may better define this area of diagnostic nomenclature in the future. In the meantime, the following suggestions to improve diagnostic precision in the DSM appear achievable.

The next revision of the DSM could benefit by: 1) extending ODD to include adults with irritability and dysfunctional anger since an arbitrary age cut-off doesn't fit NCS-R data 2) removing irritability as a definer for depressive disorders in youth, but considering it as a possible descriptor in all age groups. 3) removing irritability as a *definer *for manic episodes, since it isn't a central feature of manic mood. 4) including dysfunctional anger in the DSM as one consequence of alcohol and hallucinogenic abuse, since this is a very common occurrence 5) removing IED from Impulse Control Disorders, Not Elsewhere Classified and including it within an appropriate category, like a new dysfunctional social behavior disorder category 6) limiting the frequently used but non-specific term 'irritability' in the DSM, and--when appropriate--describing the problem as dysfunctional anger 7) expanding the term mood to apply to dysfunctional anger and anxiety as well as to depression and mania, and 8) forming an age-related continuum between conduct disorder-- callous unemotional subtype-- in adolescence and antisocial personality disorder in adulthood.

## Competing interests

The author declares that they have no competing interests.
